# Maximizing lipid accumulation in *Tetradesmus obliquus* under heavy metal stress for sustainable biodiesel innovation

**DOI:** 10.1186/s12896-025-00951-z

**Published:** 2025-03-03

**Authors:** Eman A. Alwaleed, Hamdy R. M. Galal, Mohamed Aboueldahab, Hani Saber

**Affiliations:** 1 Botany and Microbiology Department, Faculty of Science, South Valley University, Qena, 83523 Egypt; 2 State Key Laboratory of Environmental Geochemistry, Institute of Geochemistry, Chinese Academy of Sciences, Guiyang, 550081 China; 3 University of Chinese Academy of Sciences, Beijing, 100049 China

**Keywords:** Biodiesel, Heavy metals, Fatty acid, Lipid, *Tetradesmus obliquus*

## Abstract

This study investigates the potential of *Tetradesmus obliquus* for lipid accumulation under heavy metal stress and evaluates it’s aviability for biodiesel production. We surveyed how different concentrations of heavy metals, including manganese (Mn), cobalt (Co), and zinc (Zn), influence the carbohydrate & protein, lipid yield, and fatty acid profiles of *T. obliquus* cultures. Our results demonstrated that while **lipid content** increased under heavy metal stress, the extent of accumulation was highly dependent on metal type and concentration. Notably, the algal culture treated with 0.04 mM Co²⁺ showed the highest lipid accumulation. Treatment with 0.3 mM Zn²⁺ resulted in the highest proportion of saturated fatty acids (SFA). The Relative Enrichment Efficiency Coefficient (REEC) analysis demonstrated that 0.04 mM and 0.07 mM Co²⁺ led to the highest lipid and carbohydrate content stimulation. Additionally, GC-MS analysis revealed increased monounsaturated fatty acids (MUFA) under several metal stress conditions. The study demonstrated that exposure to specific concentrations of heavy metals can significantly enhance lipid accumulation and alter the fatty acid profiles of *T. obliquus*, which are crucial for improving biodiesel quality. The implications of these findings suggest that heavy metal-induced stress could be a feasible approach to enhancing lipid accumulation **for** sustainable biodiesel production, and *T.obliquus* is a promising candidate for future biodiesel production.

## Introduction

**Fig. 1:**
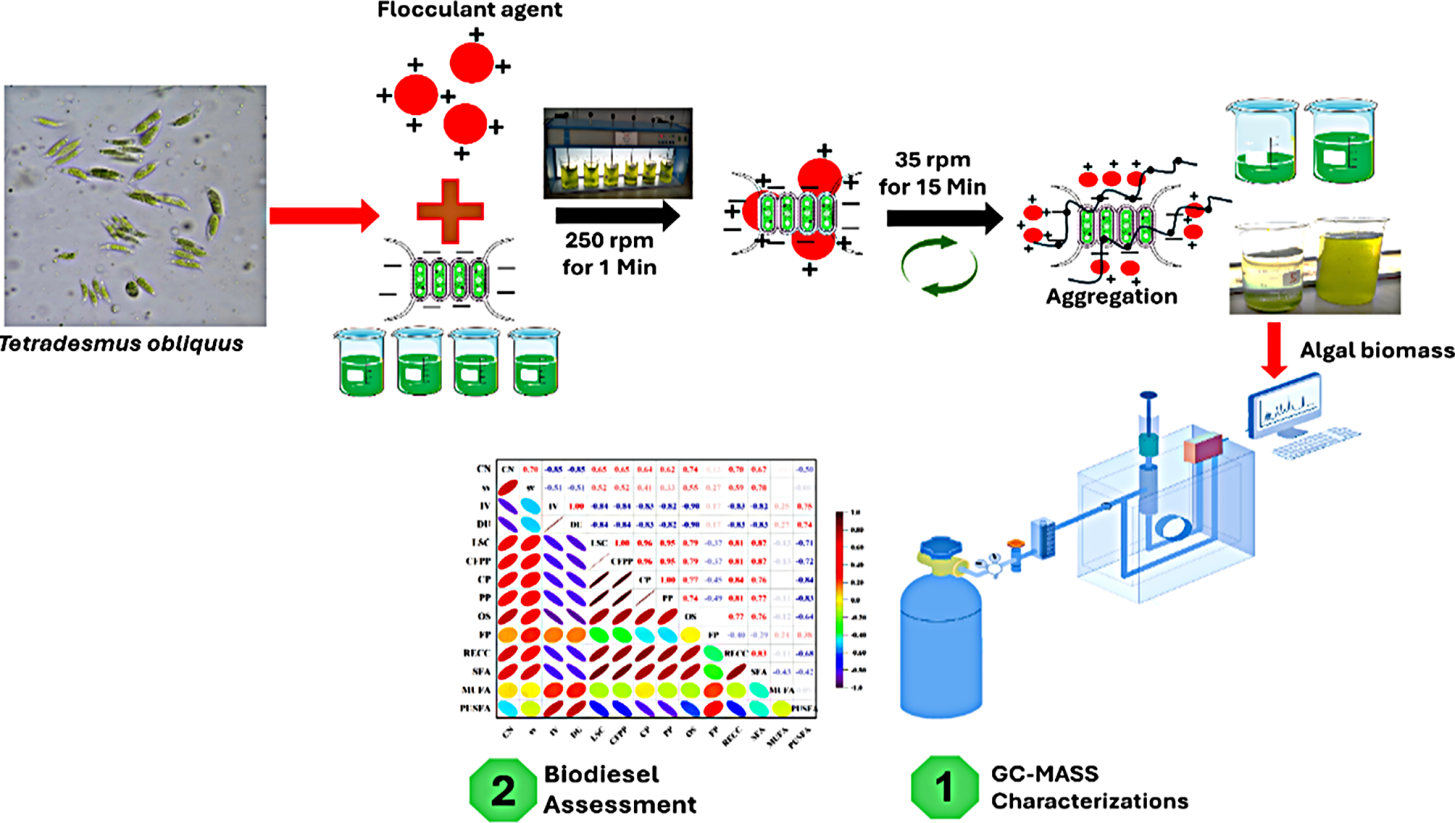
Graphical abstract of algal biodiesel production by heavy metal stress

In an era of escalating environmental challenges and a global shift toward renewable energy, microalgae have been issued as an auspicious bioresource for sustainable biodiesel production. Biodiesel is widely recognized as an eco-friendly and renewable disjunctive to conventional diesel, offering reduced greenhouse gas emissions and a sustainable pathway for energy production by utilizing natural oils and fats while maintaining similar traditional fuel efficiency [1–3]. Microalgae have recently emerged as a source of biodiesel production [4, 5] when compared to terrestrial crop-based biofuels, microalgal-based biofuels give alternative economic and environmental benefits: (i) continual development without the application of pesticides or herbicides in any environment, (ii) growth in a wide range of salt stress, (iii) high construction yields, and (iv) photosynthetic efficiency [6]. In a biorefinery-based production, residual biomass that is low in lignin but high in proteins and other commercially valuable compounds can be utilized to produce animal feed and to biosynthesize a variety of high-value chemicals, including those used to make medicines, cosmetics, and dietary supplements [7], They also evaluated for their ability to remove toxic metals from several sources [8], because they serve as essential vitamin precursors. Despite this, excessive levels of heavy metals can impair algal growth and chlorophyll production, alter photosynthetic activity, and lead to the production of reactive oxygen species (ROS) and lipid peroxidation in algal cells [9]; this disrupts membrane functions and has a detrimental effect on the cells [10]. Algal cells’ oxidized proteins and lipids may indicate that numerous microalgal organisms are under stress [11]. Various environmental stress situations have shown green microalgae to be associated with higher cellular lipid accumulation [2, 12]. High lipid content is essential for biofuel production to be commercially viable [13, 14]. The specific heavy metal and the algal species involved [15] influence the variation in the lipid content. The inhibition of the photosynthetic system, which shifts the synthesis from carbohydrates to lipids as storage molecules, can alter algal metabolic pathways. This shift may result in elevated lipid accumulation when algae are susceptible to high amounts of heavy metals [16]. *Tetradesmus obliquus* is a freshwater microalga known for its potential in biofuel production, particularly biodiesel, attributable to its high lipid content. It has gained attention for its fast growth rates and adaptability to various environmental conditions, making it a promising candidate for large-scale cultivation [17]. Effective methods for isolating algal biomass from culture media are crucial for using *T. obliquus* as a biodiesel feedstock, including moisture, salinity, cell destruction, and strain characteristics, while harvesting strategies must be regulated [18, 19]. This study demonstrates that *T. obliquus* has significant potential for enhanced lipid accumulation under heavy metal stress, which could be leveraged for biodiesel production. The qualities of biodiesel fuel were examined empirically utilizing FAME content, and the requirements of global biodiesel standards were met, as illustrated in Fig. 1. While heavy metal exposure, particularly to cobalt, increases lipid synthesis, determining the ideal metal concentration to maximize lipid yields without limiting primary parameters consider an important challenge. Furthermore, issues of scalability, metal toxicity, and lipid extraction efficiency must be addressed for large-scale industrial applications.

## Materials and methods

### Experimental layout

The microalga *Tetradesmus obliquus* (multicellular, Chlorophyta) was chosen to investigate lipid accumulation as a potential bioresource for biodiesel production, particularly under conditions of heavy metal stress, to enhance biofuel quality. The strain was isolated from the Nile River in Qena, Egypt. Before inoculating into sterile liquid nutrient media, the algal cells were separated, transferred to fresh solid media, and sub-cultured multiple times on solid BG-11 medium [20] to ensure purity.

For the experimental setup, 80 mL of the *T. obliquus* culture was introduced into a transparent, sterilized polyethylene tank with an 8-liter capacity containing BG-11 medium. The culture was incubated at 25 °C, and aeration was maintained through 3-mm polyethene tubing connected to a supply of oil-free compressed air. Illumination was provided by cool white fluorescent lights (TOSHIBA FL 40 T9D/38) under a 16:8 light-dark cycle, with a light intensity of 5000 lux, for two weeks.

The study assessed the impact of various concentrations of heavy metals, including manganese chloride (0.2, 0.4, and 0.6 mM), cobalt nitrate (0.04, 0.07, and 0.1 mM), and zinc sulfate (0.1, 0.2, 0.3, and 0.4 mM), on primary metabolites, lipid content, and fatty acid profiles.

#### Assessment of carbohydrates and protein

The Anthrone sulfuric acid technique was used to estimate carbohydrates [21, 22], and Yemm and Willis [23] adapted it. Protein content was indomitable by Lowery [24].

#### Lipid extraction

The standard *Folch method* [25] has been modified to improve lipid extraction efficiency using ultrasonic technology. One gram of dried algal biomass was homogenized in 1:2 chloroform-methanol in a glass homogenizer. After 2 min of washing with 0.9% (w/v) NaCl, the mixture was agitated at room temperature for 4 h. Mix 1 mL chloroform and 1 mL distilled water for 30 s after stirring. After that, the algal biomass and solvent mixture were sonicated in a water bath at 20–40 kHz for 15 min to improve lipid extraction. Three layers were separated by centrifuging the suspension (12,000 rpm, 4 °C, 10 min). The 0.9 % methanol/NaCl solution was discarded. Removed and stored chloroform. Again, leftover biomass was removed. Mixed chloroform extracts were stored at 4 °C overnight. A blended chloroform extract was found. Volume removes pigments and lipoproteins. Lipid extracts in weighted glass vials were dried under an argon stream, heated at 80 °C for 30 m, chilled in a desiccator, and weighted [26]. Total lipid content was calculated using formula (1):

1 $${\text{Total lipid }}\left( {\text{\% }} \right) = \frac{{{\text{lipid weight }}}}{{{\text{Algae total weight}}}} \times 100{\text{ }}$$

##### Transesterification

With 20% w/v ethanolic KOH, isolated lipids were saponified overnight at room temperature. Acidification with 5 N hydrochloric acid and petroleum ether extraction at 40–60 °C released fatty acids from potassium salts. The ether extract with fatty acid methyl esters was dried overnight with anhydrous sodium sulfate after repeated distilled water cleansing [27].

#### Trans-esterified algal oil characterization using GC–MS

The fatty acid profile was analyzed using a Trace GC1310-ISQ mass spectrometer. Retention duration and mass spectra were compared to WILEY 09 and NIST 11 databases to identify components. The most significant fatty acid mass-to-charge ratio peaks were compared to a mass spectrum database [28]. Fatty acid amounts were calculated using Eq. (2)

2 $${\text{Fatty acid proportion}} = \frac{{\text{F}}}{{{\text{Ft}}}} \times 100$$

***F*** is the desired fatty acid’s peak area, and ***F***_***t***_ is the total peak area of the fatty acid methyl ester.

#### Fatty acid patterns can be used to forecast the qualities of fuel

Predictive models utilizing FAME profiles reduce the necessity for substantial quantities of oil and specialized equipment to assess biodiesel’s chemical and physical qualities. This objective has been tackled by numerous fatty acid composition formulas [29, 30] to forecast the properties of chemical fuels, including cetane number (CN), saponification value (SV), iodine value (IV), degree of unsaturation (DU), long chain saturation factor (LCSF), cold filter plugging point (CFPP), oxidative stability (OS) [31], cloud point (CP), pour point (pp) [32], and flash point—biodiesel standards such as ASTM D6751 in the United States and EN 14214 in Europe [33].

$${\text{OS }} = {\text{ }} - 0.0384{\text{ }} \times {\text{ DU }} + {\text{ }}7.770$$

Cold flow features like cloud point (CP) and pour point (PP) values were anticipated.

The lowest temperature, or flash point (FP), was determined by Agarwal [34].

#### Selecting a promising stimulating heavy metal

Deciding on a potential heavy metal that promotes *T. obliquus* depends on biomass output and lipid and carbohydrate productivity. The same heavy metal may not induce high lipid production as high carbohydrate productivity. The selection was calculated using a unique equation based on carbs and fats’ Relative Enrichment Efficiency Coefficient (REEC, %) [35].

3 $$[(\frac{{{p_H} - {p_L}}}{{{p_L}}}){\text{ }}{\mathbf{carb}}.{\text{ }}-{\text{ }}(\frac{{{p_H} - {p_L}}}{{{P_L}}}){\text{ }}{\mathbf{lip}}.] \times 100$$

***P***_***L***_ stands for the species with the lowest productivity across all species under study, whereas ***P***_***H***_ reflects the productivity of carbohydrates (Carb.) and lipids (Lip.) for the tested species.

### Statistical analysis

Fatty acid profiles can predict fuel quality. The mean and SD from three replications are shown. The data were statistically examined using SPSS to determine significance using a one-way analysis of variance (ANOVA), LSD, and Duncan test at 0.05.

## Results

*Tetradesmus obliquus* cultures were exposed to a range of heavy metal concentrations, including MnCl_2_ (0.2, 0.4, and 0.6 mM), Co(NO_3_)_2_.6 H_2_O (0.04, 0.07, and 0.1 mM), and ZnSO_4_.7H_2_O (0.1, 0.2, 0.3, and 0.4 mM), to determine their effects on the concentration of carbohydrates, proteins, lipids and the fatty acid profile.

### Lipid contents

**Fig. 2:**
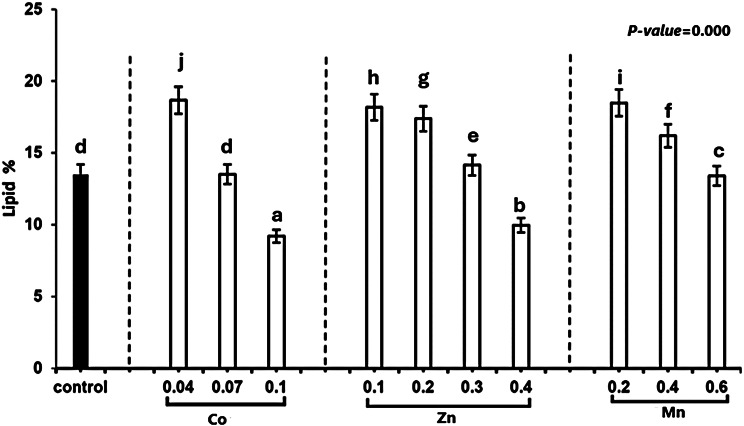
Effect of different concentrations of Cobalt nitrate, Manganese chloride and Zinc sulfate on the lipid content of *Tetradesmus obliquus* cultivated for 13 days

**Fig. 3:**
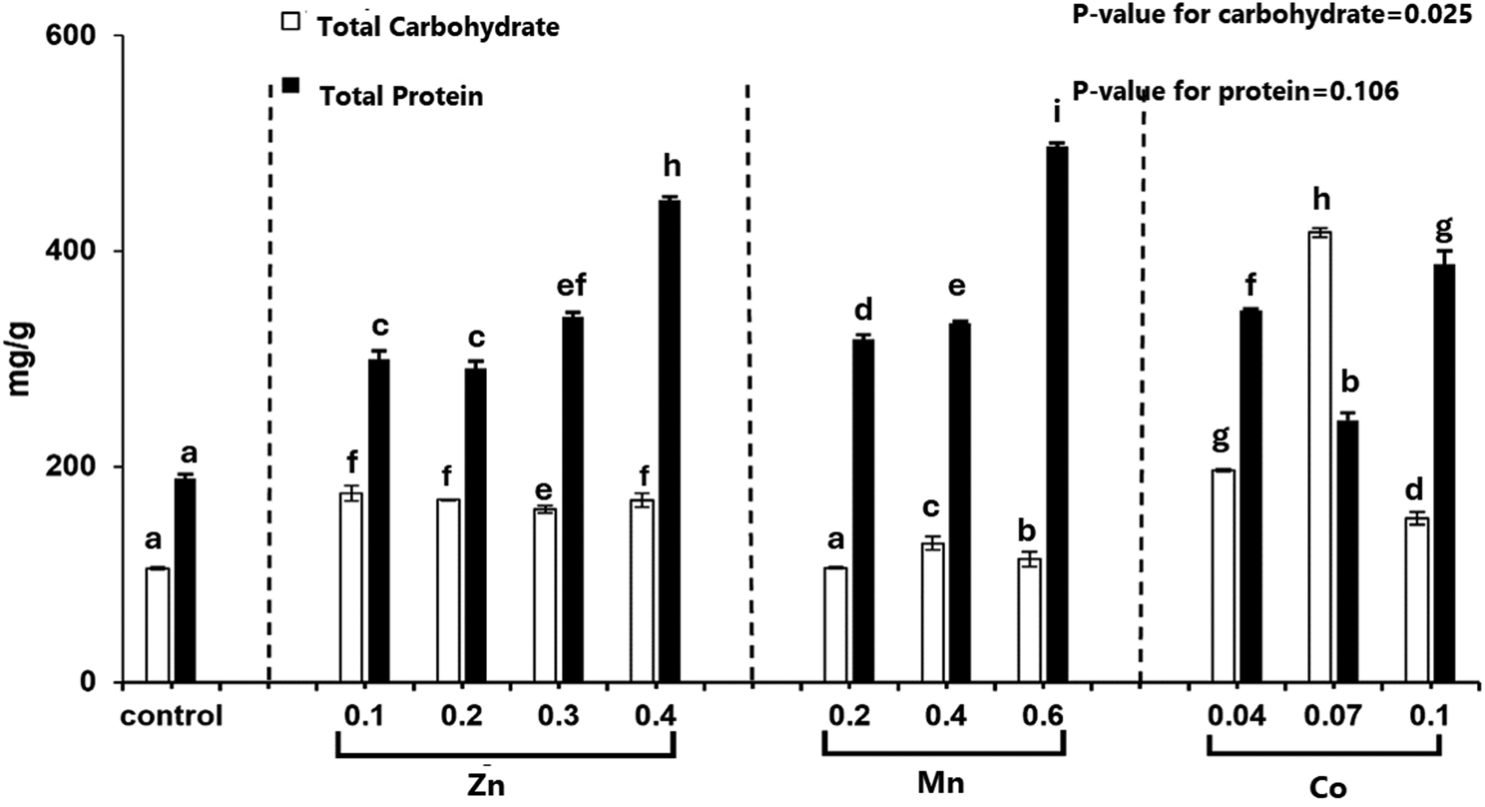
Effect of different concentrations of Manganese chloride, Zinc sulfate, and Cobalt nitrate on total carbohydrate and total protein content of *Tetradesmus obliquus* cultivated for 13 days

From the results in Fig. 2, It is possible to conclude that using varied heavy metal concentrations causes a significant alteration in the lipid content of *T. obliquus*. The maximal lipid accumulation in the 0.04 mM Co^2+^ culture was 180.67 mg/g dry wt. At the end of the experimental period, the total lipid was (180.48, 160.19, 130.34, 180.67, 130.51, 90.19, 180.18, 170.38, 140.14, and 90.96) mg/g dry wt, at the heavy metal concentrations of manganese chloride (0.2 mM, 0.4 mM, 0.6 mM), cobalt nitrate (0.04 mM, 0.07 mM, and one mM) and ZnSO_4_.7H_2_O (0.1, 0.2, 0.3, and 0.4 mM) respectively. Combining factors such as lipid and carbohydrate profitability, (REEC,%) showed that 0.04 and 0.07 mM Co^2+^ led to significant increasing in lipid and carbohydrate content in treated *T. obliquus* by compared to the control. At 0.189 and 0.341 × 10^3^ REEC %, where carbohydrates reached 196.83 and 417.29 mg/g compared with control at 105.64 mg/g, as illustrated in Fig. 3, and lipids increased to 18.98 and 13.52 % DW compared to 13.51 % DW in control. Therefore, *T. obliquus* could be selected as a promising microalga for additional research as a dual bioethanol and biodiesel producer.

### Mass spectrometry using gas chromatography (GC/MS)

**Fig. 4:**
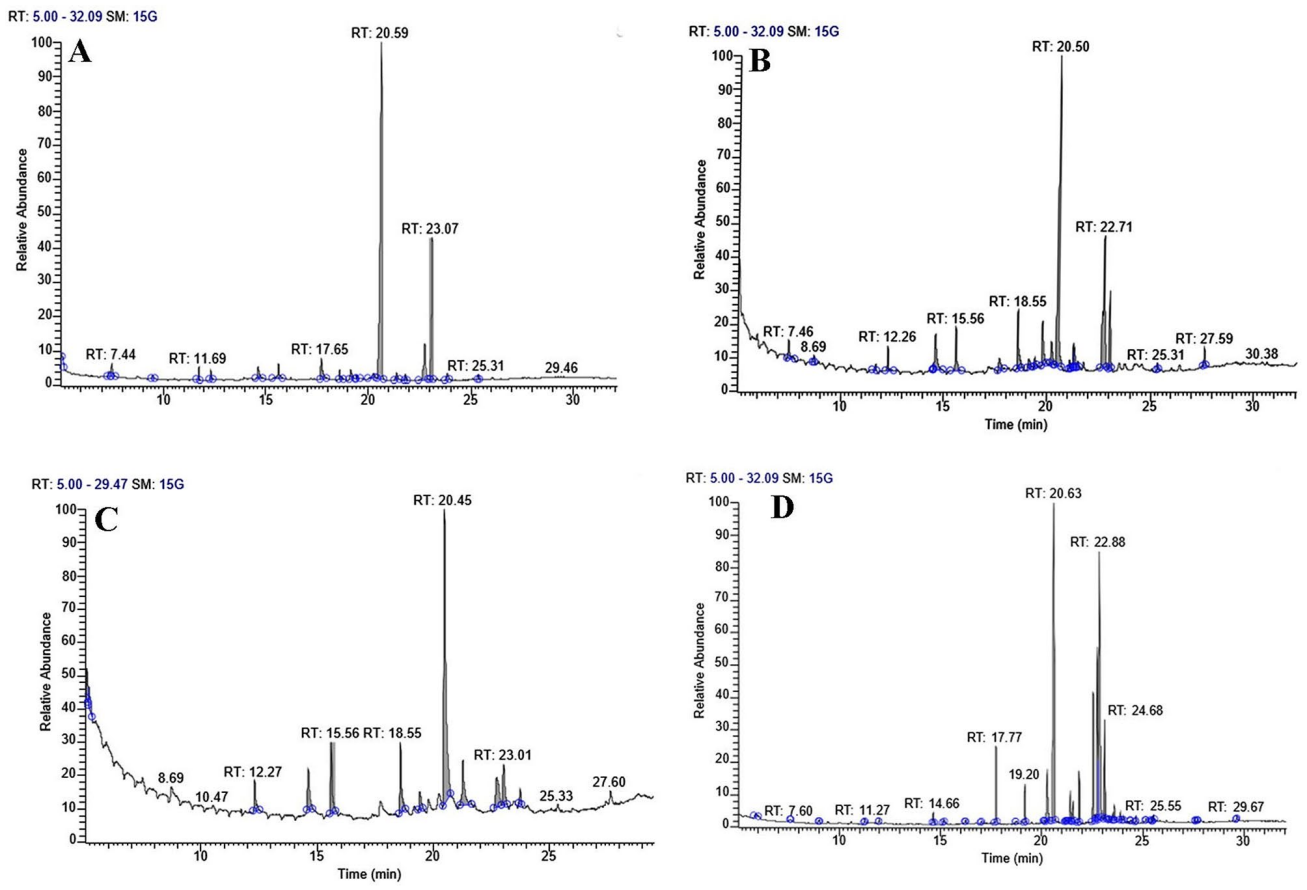
GC-Mass chromatogram of *Tetradesmus obliquus* treated with the different concentrations of manganese chloride: **A** Control, **B** 0.2 mM Mn, **C** 0.4 mM Mn and **D** 0.6 mM Mn after transesterification process

**Fig. 5:**
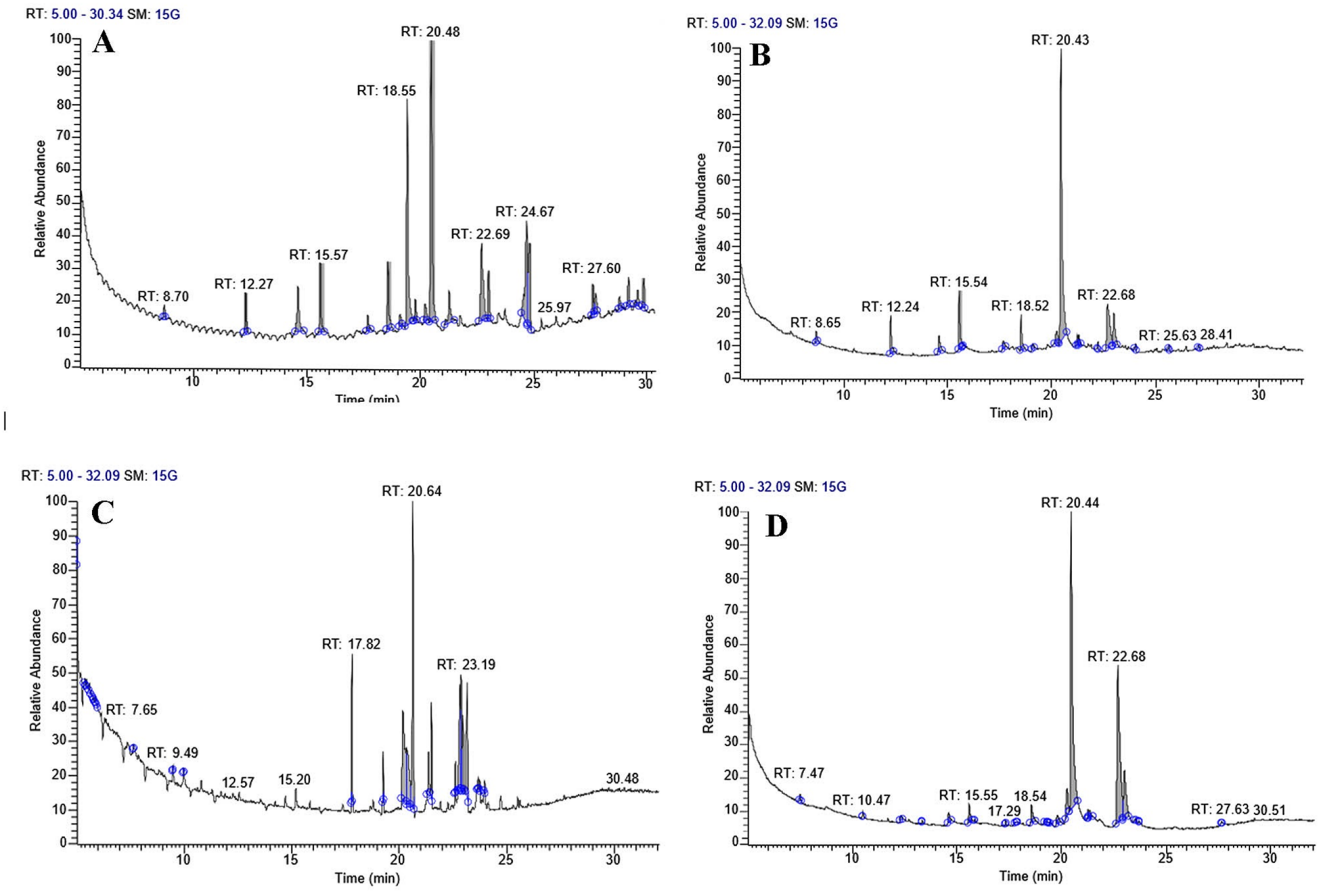
GC-Mass chromatogram of *Tetradesmus obliquus* treated with the different concentrations of Zinc sulfate **A** 0.1 mM Zn, **B** 0.2 mM Zn**, C** 0.3 mM Zn, and **D** 0.4 mM Zn after transesterification process

**Fig. 6:**
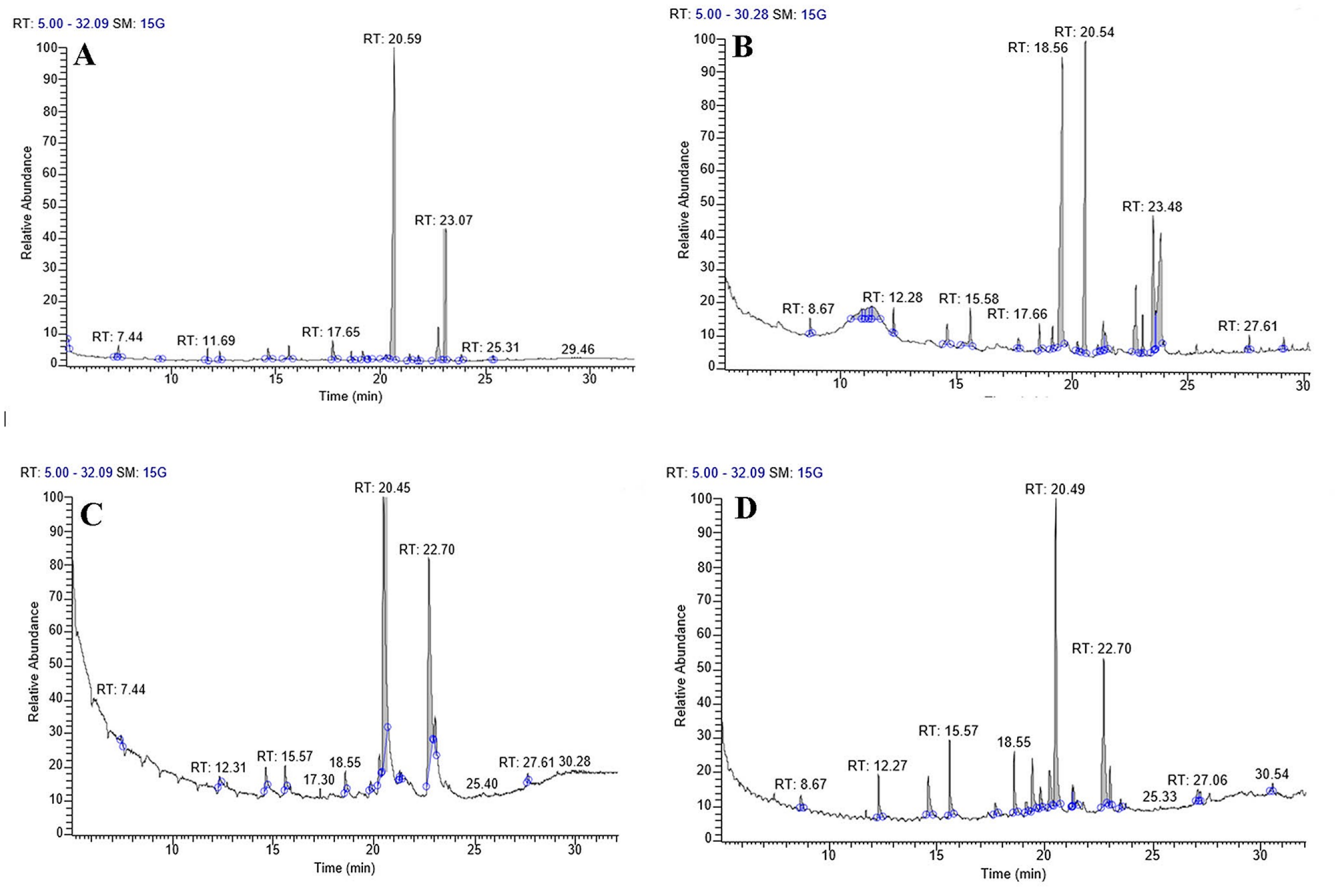
GC-Mass chromatogram of *Tetradesmus obliquus* treated with the different concentrations of Cobalt nitrate **A** Control, **B** 0.1 mM Co, **C** 0.04 mM Co, and **D** 0.07 mM Co after transesterification process

A mobile phase is used in the separation process known as chromatography, carrying the mixture through a selective absorbent stationary phase. It controls and standardizes phytocomponent quality. The test algal extract components’ names, molecular weights, and structures were determined by GC–MS. It is mainly used to determine thermochemical constants, purify chemicals, and perform qualitative and quantitative analyses on mixtures. The chemical composition of algal extracts was analyzed through GC–MS following lipid and fatty acid esterification, revealing the presence of various compounds. The fatty acid profiles of *T. obliquus* exposed to different concentrations of heavy metals were assessed during the stationary growth phase, as illustrated in Figs. 4, 5 and 6. The predominant fatty acids were palmitic acid C18:0 and stearic acid C16:0, and their concentrations varied noticeably with varying levels of heavy metals. Treatment with Zn^2+^, Co^2+,^ and Mn^2+^ resulted in an increase of saturated fatty acids contents (SFA) except for (0.4 mM Zn^+2^), (0.04, 0.1 mM Co^+2^) and (0.4 and 0.6 mM Mn^2+^) which show decreases in comparison to control. Meanwhile, monounsaturated fatty acids (MUFA) increased under all heavy metal concentrations. In contrast, total PUFA decreased under the effect of all different heavy metal concentrations. Besides that, the result showed that a high percentage of total SFA characterizes the control culture of *T. obliquus,* reaching 60.14 % due to the presence of C4:0, C10:0, and C18:0 by 26.62, 8.33, and 19.52 %, respectively in comparison to low amount of MUFA (9.82%) and highly amount of PUFA (27.18%) due to presence of linoleic acid. The highest saturated fatty acid (SFA) content, amounting to 83.62%, was attributed to the presence of myristic acid (C14:0), palmitic acid (C16:0), and stearic acid (C18:0), which constituted 3.75%, 58.66%, and 15.69% of the total fatty acids, respectively in cultures treated with 0.07 mM Co²⁺. Additionally, the application of various heavy metal concentrations significantly increased the proportion of SFA to 64.77%, 65.67%, 76.24%, 83.62%, and 65.46% of the total fatty acids at 0.1, 0.2, and 0.3 mM ZnSO₄, 0.1 mM Co(NO₃)₂ · 6H₂O, and 0.2 mM MnCl₂, respectively. The highest levels of monounsaturated fatty acids (MUFA) were observed at 49.29%, 38.11%, 31.81%, and 30.73% in algal cultures treated with 0.04 mM Co²⁺, 0.4 and 0.2 mM Zn²⁺, and 0.6 mM Mn²⁺, respectively, predominantly due to the presence of oleic acid (C18:1 n-9) and palmitoleic acid (C16:1 n-7). Research shows that palmitic acid (C16:0) and stearic acid (C18:0) are the most abundant saturated fatty acids (SFA), while palmitoleic acid (C16:1 n-7) and oleic acid (C18:1 n-9) are the most prevalent MUFA. Among the polyunsaturated fatty acids (PUFA), linoleic acid (C18:2 n-6), stearidonic acid (C18:4 n-3), and arachidonic acid (C20:4 n-6) are the highest dominant. Notably, increasing concentrations of heavy metals have been shown to promote MUFA production, though saturated fatty acid accumulation tends to rise under most levels of heavy metal exposure. Finally, adding heavy metals to the *T. obliquus* fatty acid profile improves the saturated fatty acid (SFA) and mono-saturated fatty acid (MSFA) in the treated culture compared to the control culture, which increasing the quantity and quality of biodiesel.

**Fig. 7:**
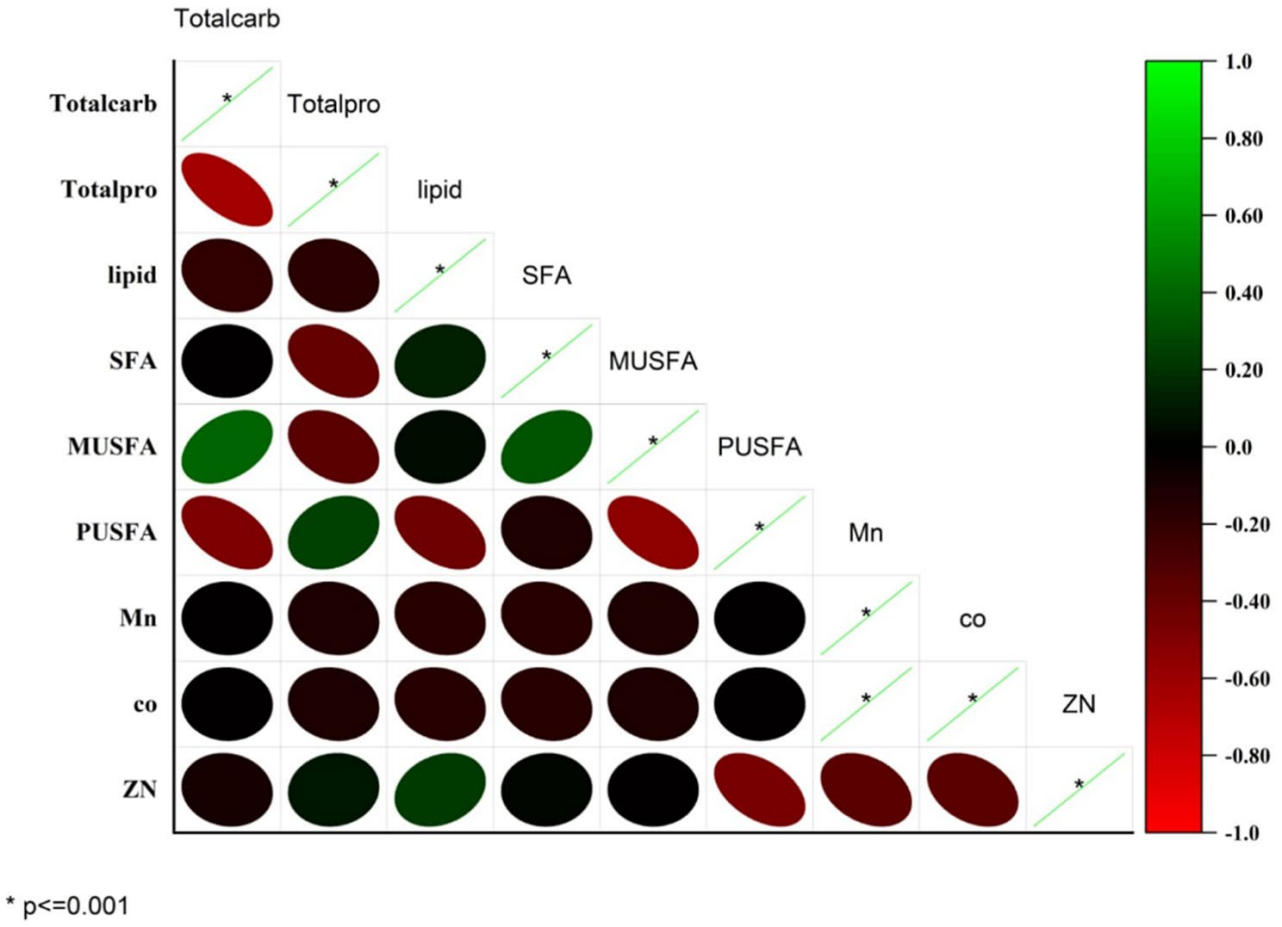
A heat map illustrates the Pearson correlation between the carbohydrate, protein, and fatty acid profile of *Tetradesmus obliquus* under the different concentrations of heavy metal

As demonstrated in Fig. 7 The Pearson correlation matrix highlights significant physiological and biochemical interactions in *T. obliquus*, revealing a significant negative correlation between total protein and lipid content (−0.357*, *p* < 0.05) suggests a metabolic tradeoff, where increased protein synthesis may occur at the expense of lipid storage, reflecting resource allocation priorities under varying conditions. Similarly, a strong negative correlation between saturated fatty acids (SFA) and monounsaturated fatty acids (MUSFA) (−0.692*, *p* < 0.01) indicates a tightly regulated balance, where the synthesis of one type of fatty acid inhibits the other, potentially driven by enzymatic competition in lipid metabolism. Total carbohydrate shows weak correlations with other physiological factors and heavy metals, suggesting a relatively independent pathway or minor influence under the studied conditions. Heavy metals exhibit non-significant correlations with physiological components. These findings collectively highlight the complex metabolic interplay within *T. obliquus*, emphasizing the significance of tradeoffs between protein-lipid and fatty acid dynamics, while suggesting that heavy metal concentrations may play a secondary role in shaping these physiological processes.

### Predicting biodiesel quality metrics from the content of fatty acids

The structure and composition of fatty acids play a fundamental role in determining the properties and quality of biodiesel. In addition to the untreated control culture, *T. obliquus* treated with several heavy metals Co (NO_3_)_2_.7H_2_O, MnCl_2_, and ZnSO_4_.6H_2_O, as illustrated in Table 1, were determined to have ten essential biodiesel qualities; values are assumed as percent of total fatty acids, also the saturated fatty acid (SFA) and unsaturated fatty acid (UFA) were Not detected (ND). Table 2 lists the predicted qualities together with the matching European (EN 14214), American (ASTM D6751), and Indian (IC15607) biodiesel standards.

**Table 1:** Fatty acid profiles of *Tetradesmus obliquus* treated with different concentrations of heavy metals

Lipid Number	Control	Zn (Mm)				Co (mM)			Mn (mM)		
		0.1	0.2	0.3	0.4	0.04	0.07	0.1	0.2	0.4	0.6
C3:0	ND	ND	ND	ND	ND	ND	ND	5.67	ND	ND	ND
C4:0	26.62	ND	ND	ND	ND	ND	0.64	ND	ND	2.49	ND
C6:0	ND	16.74	ND	ND	ND	ND	ND	ND	ND	ND	ND
C10:0	8.33	ND	ND	ND	ND	ND	ND	ND	ND	ND	0.17
C11:0	ND	ND	ND	ND	ND	ND	ND	ND	ND	ND	ND
C12:0	0.82	18.95	ND	ND	0.02	ND	0.71	ND	ND	ND	5.30
C13:0	ND	ND	ND	ND	ND	ND	ND	ND	ND	ND	0.08
C14:0	0.65	1.11	ND	6.75	0.10	ND	3.75	3.13	8.76	ND	3.89
C15:0	ND	ND	ND	1.90	1.30	ND	1.90	1.53	1.38	ND	1.89
C16:0	19.52	21.73	56.69	52.69	44.67	43.32	58.66	43.92	41.13	43.49	30.55
C17:0	1.04	ND	ND	ND	9.27	ND	1.41	ND	6.48	5.96	3.80
C18:0	1.82	3.03	7.28	11.74	0.03	0.42	15.69	2.14	6.63	ND	5.67
C19:0	ND	ND	ND	ND	ND	ND	ND	ND	ND	ND	0.27
C20:0	1.34	ND	ND	3.16	ND	ND	0.50	ND	0.48	7.71	0.18
C22:0	ND	2.94	1.70	ND	0.15	ND	ND	ND	ND	ND	0.24
Σ SFA	**60.14**	**64.77**	**65.67**	**76.24**	**55.54**	**43.74**	**83.62**	**59.80**	**65.46**	**59.65**	**50.85**
C16:1n-7	3.62	7.86	12.54	5.30	4.41	7.15	5.39	5.51	3.36	11.05	5.39
C18:1n-9	6.20	6.05	19.28	13.73	33.68	42.14	5.62	19.62	20.12	8.59	25.34
C20:1	ND	ND	ND	0.36	0.02	ND	1.92	ND	ND	ND	ND
Σ MUSFA	**9.82**	**13.91**	**31.81**	**19.39**	**38.11**	**49.29**	**12.93**	**25.13**	**23.50**	**19.64**	**30.73**
C18:2N-6	27.10	8.96	1.58	9.21	0.12	0.68	2.48	1.17	ND	8.52	20.20
C18:4n-3	ND	ND	ND	ND	ND	ND	ND	ND	ND	ND	ND
C20:4n-6	ND	ND	ND	ND	ND	ND	ND	ND	ND	ND	ND
Σ PUSFA	**27.18**	**8.96**	**1.58**	**9.21**	**0.12**	**0.68**	**2.48**	**1.17**	**ND**	**8.52**	**20.20**
TFA	**97.14**	**87.39**	**99.06**	**95.63**	**93.77**	**93.71**	**99.05**	**86.10**	**88.96**	**87.81**	**100**

*ND* ‘Not Detected’. Bold numbers illustrated the Σ SFA, Σ MUSFA, Σ PUSFA and TFA

**Table 2:** Predicted biodiesel properties from FAME profiles of Scenedesmus and specifications are in U.S., European and Indian standards

Heavy metals	Concentration	CN	SV (mg KOH g^-1^)	IV (g I_2_ 100 g^−1^ fat)	DU (wt., %)	LCSF (wt., %)	CFPP(°C)	CP(°C)	PP(°C)	OS (hr.)	FP (min.)	REEC *10^3^
	Control	61.52	196.99	55.29	64.18	2.09	−9.93	5.275	−1.10	5.31	191.35	0.047
Zn (mM)	0.1	69.10	232.51	28.05	31.83	3.69	−4.89	6.44	0.167	6.55	191.49	0.163
	0.2	66.03	203.54	31.24	34.97	15.50	32.21	24.83	20.13	6.43	185.42	0.149
	0.3	64.78	210.34	32.97	37.81	13.83	26.96	22.72	17.85	6.32	178.25	0.105
	0.4	67.48	189.34	33.70	38.35	4.707	−1.69	15.51	13.27	6.30	189.55	0.060
Co (mM)	0.04	66.45	186.00	40.63	50.65	4.54	−2.21	17.80	12.50	5.83	183.77	0.189
	0.07	69.58	203.12	15.75	18.71	13.71	26.60	25.87	21.26	7.05	179.00	0.341
	0.1	68.35	198.00	24.30	27.64	9.17	12.34	18.11	12.84	6.71	191.34	0.044
Mn (mM)	0.2	72.29	178.36	20.27	23.50	7.91	8.37	16.65	11.25	6.87	185.72	0.102
	0.4	68.80	182.65	32.56	36.68	12.06	21.40	17.89	12.60	6.36	200.89	0.098
	0.6	63.46	169.16	66.86	70.74	5.43	0.58	11.05	5.20	5.05	179.32	0.064
Biodiesel standard EN 142214		≥51	-	≤120	-	-	≤5/-20	-	-	≥6	120	
Biodiesel standard ASTM D6751-02		≥47	-	NA	-	-	NA	-	-	≥6	93	
Biodiesel standard IC15607		≥51	-	NA	-	-	6/18	-	3/15	≥6	-	

*CN* Cetane number, *SV* Saponification value, *IV* Iodine value, *DU* Degree of unsaturation, *LCSF* Long chain saturated factor, *CFPP* cold filter plugging point, *CP* Cloud point, *PP* Pour point, *OS* Oxidative stability, *HHV* Higher heating value, *FP* Flashpoint

The cetane number (CN), analogous to the octane number for gasoline, is a dimensionless metric that evaluates a fuel’s ignition quality in a diesel engine. An elevated CN value may exist due to the substantial quantity of saturated and monounsaturated methyl esters. The present investigation indicates that all *T. obliquus* specimens treated with various heavy metals exhibited elevated CN levels, ranging from 63.46 to 72.29. The control culture achieved a cetane number (CN) of 61.52, surpassing the European biodiesel standard EN 14214, which mandates a minimum CN of 51, the Indian biodiesel standard requiring at least 47, and the American Petro-diesel standard ASTM D97550, which stipulates a minimum CN of 40. The fatty acid profile analysis indicated a significant concentration of saturated fatty acids (SFA), predominantly palmitic acid (C16:0), comprising 19.52 to 58.66% of total fatty acids (TFAs), and stearic acid (C18:0), accounting for 0.03 to 15.69% of TFAs, which lacks unsaturated linkages between carbon atoms and therefore cannot accommodate additional hydrogen atoms. The results indicated that the 0.2 mM Mn culture exhibited the highest CN value (72.29), succeeded by cultures treated with 0.07 mM Co^2+^ (69.58) and 0.1 mM Zn^2+^ (69.58). The saponification value of treated and untreated *T. obliquus*, based on the fatty acid profile data, varied from 169.16 mg KOH g⁻¹ in the control culture to 203.54 mg KOH g⁻¹ in the treated culture with 2 mM MnCl₂.

The saponification value is the milligrams of KOH needed to neutralize one gram of oil’s fatty acids. The findings revealed that both treated and untreated *T. obliquus* exhibited reduced iodine values, ranging from 15.75 g I_2_/100 g at 0.07 mM Co(NO_3_)_2_ · 6H_2_O to 6.86 g I_2_/100 g at 0.6 mM MnCl_2_. The data indicate that applying several heavy metal stressors on *T. obliquus* significantly increases the SV value of the generated biodiesel. The iodine value quantifies the degree of unsaturation in fatty acids. Consequently, treated and untreated *T. obliquus* biodiesels comply with the maximum iodine value limitations (120 g I_2_/100 g) established by ASTM D6751, EN 14214, and IS 15607 standards. The lowest IV is associated with biodiesel containing minimal unsaturated fatty acids.

The biodiesel with the lowest IV has the fewest unsaturated fatty acids. The degree of unsaturation (DU) was calculated by summing the masses of MUFA and PUFA, which measure unsaturated FAs. It’s crucial to biodiesel’s oxidative stability. PUFA were abundant in biodiesel due to their high unsaturation, which reduced its oxidative stability. FAME DU from all treated *T. obliquus* ranged from 18.71 wt.% to 0.07 mM Co. (NO_3_).6H_2_O to 70.74 wt. % at 0.6 mM MnCl_2_ (Table 2), below EN 142214’s maximum limit of 137 wt. Low-temperature biodiesel properties like (CFPP), (CP), and (PP) were evaluated by FA content.

Cold flow performance is crucial to biodiesel. The CFPP value in biodiesel standard EN 142214 should be within 5/−20 °C, while the CFPP value in biodiesel standard IS 15607 should be within 6/18 °C. Except for 3 M Co, most treated *T. obliquus* FAMEs match these requirements (NO_3_). CFPP was 28.33 °C in 6H_2_O. The highest temperature of CFPP might be present due to a high amount of SFA, namely Palmitic acid (C16:0), which is found at a level greater than 32.04 % in treated *T. obliquus*.

Cloud point (CP) determines the low temperature at which the solution’s least soluble biodiesel component crystallizes. In this study, the results illustrated that the estimated cloud point for treated *T. obliquus* biodiesels varied from 6.44 °C in 0.1 mM ZnSO_4_.7H_2_O to 25.87 °C in 0.07 mM Co displayed the highest cloud point due to its higher saturated fatty acids content (59.80 %).

The pour point (PP) is the lowest temperature at which the fuel freezes solid and loses its flow performance, being no longer pumpable; therefore, it is an indicator of the diesel gelling point. This study showed that PP values for tested *T. obliquus* varied from 1.10 to 21.26 °C. Most of the treated *T. obliquus* FAMEs successfully meet these criteria according to Indian standard (IS 15607) except 0.2 and 0.3 mM ZnSO_4_ and 0.07 mM, Co (NO_3_)_2_.6H_2_O which have pp values at 20.13, 17.85 and 21.26 °C, respectively.

Long-chain saturation factor affects cetane number (CN), iodine value (IV), oxidation stability (OS), and cold filter plugging point (CFPP) in biodiesel. Long-chain fatty acids precipitate at higher temperatures than shorter-chain ones; hence, a higher proportion will affect low-temperature properties. Due to its high Palmitic acid (C16:0) content, 0.2 mM Zn had the highest LCSF value (15.50 wt.%) in this study, while control had 2.09 wt. The storage stability of biodiesel, which is essential for fuel applications, is significantly influenced by large quantities of polyunsaturated FAs that oxidize quickly. This study found that the oxidative stability of treated *T. obliquus* FAME ranged from 5.05 to 7.05 h, meeting the biodiesel standard EN 142214, ASTM D6751-02, and IS 15607 EN 14214 suggested value of ≥6 h due to low PUFA content.

The flash point (FP) is the lowest temperature at which fuel vaporizes, forming an ignitable combination with air. Australian and European biodiesel regulations (EN 142214) need a flash point temperature of 120 °C, but the US standard (ASTM D6751-02) requires 93 °C. Higher flash points make fuels safer for storage, handling, and transportation without affecting combustion. The estimated FP temperature for all treated *T. obliquus* was 178.25–200.89 °C, above biodiesel guidelines. High FP made *T. obliquus* biodiesel safe for storage and transfer.

**Fig. 8:**
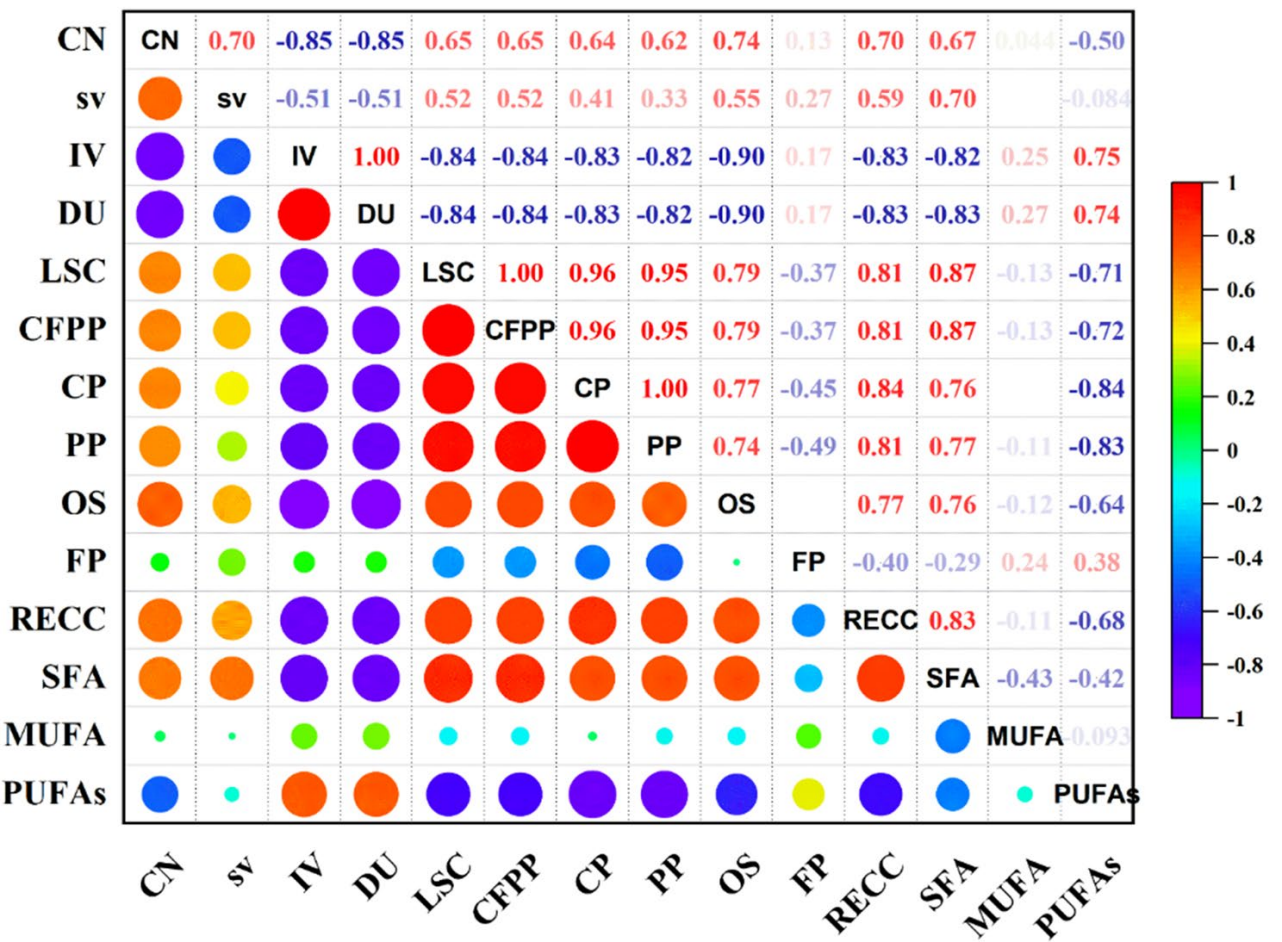
A heat map illustrates the Pearson correlation between the fatty acid profile of *Tetradesmus obliquus* and the effect of different concentrations of heavy metal and biodiesel properties

As demonstrated in Fig. 8, saturated fatty acid (SFA) of the selected alga shows positive correlation effect on CN, SV, LSCF, CFPP, CP, PP and RECC (r = 0.279, 0.406, 0.640*, 0.640*, 0.470, 0.442, 0.039 and 0.505 respectively at *p* < 0.05) in contrast Saturated fatty acid (SFA) was highly negatively correlated with IV, DU and FP respectively at (r = −0.598, −629* and −0.287* at *p* < 0.05). Monounsaturated fatty acid (MSFA) was positively correlated with SV, DU, LSCF, OS, and FP (r = 0.935, 0.183, 0.215, 0.212, and 0.528), while CN, LSCF, and CFPP negatively correlated with MUFA. SV, IV, DU, OS, and FP were affected positively by an increase of Polyunsaturated fatty acid (PUFA) (r = 0.404, 0.486, 0.467, and 0.147, respectively, at *p* < 0.05). Still, it negatively correlated with pp and CN(r = −0.638* and −0.280 at *p* < 0.05).

## Discussion

*T. obliquus* cultures were exposed to several doses of heavy metals to study the effects on metabolic activity, MnCl_2_ (0.2, 0.4, and 0.6 mM), Co(NO_3_)_2_.6H_2_O (0.04, 0.07, and 1 mM), and ZnSO_4_.7H_2_O (0.1, 0.2 mM, 0.3, and 0.4 mM) individually. The remarkable resilience of *T. obliquus* is on full display in its ability to withstand various challenging environmental factors, including heavy metal exposure. The development of *T. obliquus* was positively affected by most of the heavy metal concentrations examined. For example, increased quantities of manganese (Mn²⁺), essential for several metabolic processes, vitamins, and proteins, may have contributed to growth improvement [36]. Additionally, the replacement of Zn²⁺ with Co²⁺ in certain metalloenzymes could explain the growth promotion observed at low concentrations of Co²⁺ [37]. As reported by Cheng [38], lower concentrations of Zn²⁺ tend to stimulate algal growth. Furthermore, microalgae exhibit a homeostatic regulatory mechanism under heavy metal stress that triggers an over-compensatory response, activating metabolic and antioxidant systems to mitigate the toxic effects, commonly called the hermetic effect [39]. In addition to their potentially harmful effects, certain heavy metals may also serve as essential nutrients that support microalgae growth, complementing the existing nutrient sources in the culture medium; for example, copper (Cu²⁺) and zinc (Zn²⁺) function as vital cofactors in enzymes involved in critical processes such as mitochondrial and chloroplast function, DNA synthesis, and photosynthetic electron transport [40]. While zinc is essential for algal metabolism, excessive concentrations can be detrimental, as demonstrated by Afkar et al. [41]. When heavy metals are present in concentrations higher than the organism’s tolerance; they can disrupt cellular processes by inhibiting cell division, inactivating proteins, and causing structural disorganization of mitochondria and chloroplasts. These effects may extend to the rupture of the chloroplast envelope, the deterioration of membrane integrity, and, ultimately, cell lysis [42–44]. These disruptions explain the inhibitory effects observed at higher concentrations of heavy metals on algal growth. Carbohydrate buildup within algal cells is considered the primary organic component of photosynthetic action and crucial for biofuel production [45]. Under heavy metal stress, *T. obliquus* exhibited significant carbohydrate, protein, and lipid content alterations. The highest carbohydrate content was observed with 0.07 mM Co²⁺, reaching 417.29 mg/g, compared to 105.64 mg/g in the control. Our results were similar to Sharma & Agrawal [46], who found that heavy metals caused significant increases in the amount of carbohydrates in algal cultures. The accumulation of soluble carbohydrate fractions may mitigate heavy metal stress, which appears to be an effective approach [47]. Battah’s research (2015) [36] indicated that carbohydrate levels rose when concentrations of Mn^2+^, Co^2+^, and H_2_O_2_ levels dropped. The strategy appeared effective for producing substantial amounts of soluble carbohydrates to alleviate heavy metal stress [47].

**Fig. 9:**
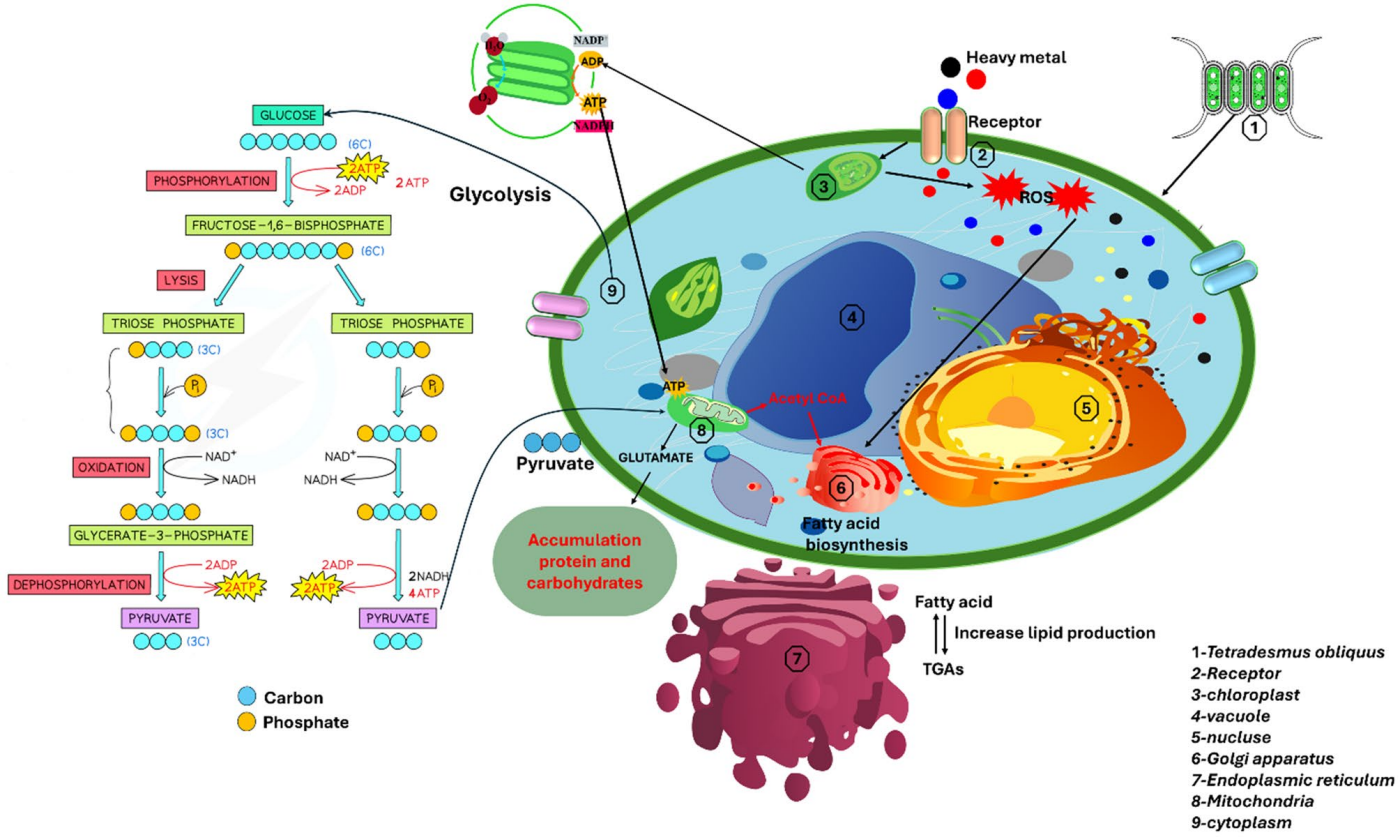
Illustrates the mechanism of increased metabolic products, including lipids, carbohydrates, protein, heavy metal stress, and reactive oxygen species (ROS) formation

The response of *T. obliquus* to different heavy metals, in terms of total protein content, varied according to the type and concentration of the heavy metal. The effects of heavy metals on *T. obliquus* revealed that protein levels fluctuated depending on the specific element and its dosage. Heavy metal exposure can elevate reactive oxygen species (ROS) levels Figure 9, leading to protein oxidation and degradation [48]. The declined total protein content in algae treated with heavy metals may be due to oxidative damage, accelerating protein degradation. However, increased total protein content under certain heavy metal stress conditions could be linked to the upregulation of stress-related proteins, particularly enzymes involved in antioxidant metabolism and the biosynthesis of photoprotective compounds [49]. It has been suggested that protein accumulation at lower concentrations of heavy metals may be one mechanism by which algae mitigate toxicity or that increased respiration leads to carbohydrate consumption, thereby promoting protein accumulation [50].

Conversely, protein accumulation may be hindered by a lack of carbon skeletons resulting from reduced photosynthetic activity [41]. Additionally, protein reduction could be attributed to protein hydrolysis into amino acids, which may serve osmotic functions [51], or decreased availability of amino acids needed for protein synthesis; this could also be due to the denaturation of enzymes involved in amino acid and protein synthesis [52].

Increased cellular lipid accumulation in green microalgae has been commonly observed under various environmental stress conditions. High lipid production is essential for the commercial feasibility of biofuel production [53]. Suitable microalgae species must exhibit high lipid productivity by maintaining a large basal lipid content or accumulating substantial quantities of lipids under stress [13]. The study shows that lipid content in *T.obliquus* increased at specific heavy metal concentrations, notably 0.2 and 0.4 mM Mn²⁺, 0.04 mM Co²⁺, and 0.1, 0.2, and 0.3 mM Zn²⁺. Lipid accumulation reached its highest level of 180.68 mg/g dry weight (CDW) under 0.04 mM Co²⁺ treatment, compared to 130.52 mg/g observed in the control. However, lipid content significantly decreased when heavy metal concentrations exceeded these levels. These findings align with Napan et al. [14], who identified an optimal concentration range for heavy metals that maximizes lipid production, with variations depending on the specific metal and algal species. Within this optimal range, heavy metal stress may promote lipid accumulation [15]. The increased lipid content observed in *T. obliquus* at higher concentrations of Co²⁺ could be due to disruptions in algal metabolism, particularly the inactivation of the photosynthetic machinery, leading to lipid accumulation as storage molecules in place of carbohydrates [54]. Heavy metals also initiate oxidative stress through the increased production of reactive oxygen species (ROS) [55]. It has been suggested that microalgae increase lipid production as a defense mechanism to sequester ROS Fig. 7, thereby accelerating the biosynthesis of scavenger molecules [56]. The stress induced by heavy metals may arise from redox-active metals like Co (II) and Mn (II), which catalyze ROS generation, or from non-redox-active metals like Zn (II), which promote the production of ROS-scavenger molecules. However, excessive heavy metal exposure can damage cellular structures such as the chloroplast and disrupt the endoplasmic reticulum, ultimately reducing lipid yields [57].

### Fatty acids composition

The essential traits for selecting algal strains for biodiesel generation are higher lipid levels and volumetric lipids [30]. Thus, the current work focused on carefully analyzing fatty acid content. The fatty acid profiles of *T. obliquus* revealed palmitic acid, palmitoleic acid, stearic acid, oleic acid linolenic acid. According to earlier studies, the primary fatty acids found in vegetable oils and other microalgae species [34, 58] include palmitic, stearic, oleic, linoleic, and linolenic. Remarkably, the most often occurring fatty acids in treated algae are palmitic acid and stearic acid. High saturated esters (SFA) presence in fuel lowers NOx exhaust emissions when compared to unsaturated esters (UFA). Higher cetane number (CN), which indicates superior oxidative stability and ignition quality of biodiesel, gives another benefit of saturated fatty esters [31]. Higher chain fatty acids, namely C18:2 and C18:3, were lower in algal oils than in vegetable oils [58, 59], supporting the preceding finding. Myristic acid, stearic acid, and palmitic acid were most remarkable in the treated *T. obliquus* strains [60], which are favourable for biodiesel production [61, 62]. Furthermore, the FA composition revealed that SFA had a higher proportion than MUFA and PUFA. Previous studies discovered that low quantities of polyunsaturated and saturated fatty acids, rather than monounsaturated, are more suited for reducing oxidative stability and cold flow difficulties [34]. It was hypothesized that cells resistant to growth inhibitors may synthesize polyunsaturated fatty acids due to the inhibitor’s influence on fatty acid desaturation [63]. The overall impact of Mn^+2^, Co^+2^, and Zn^+2^ was to raise the ratio of unsaturated to saturated fatty acids, hence enhancing membrane fluidity [34], and membrane fluidity can vary depending on the length of the fatty acid chain [64]. In our investigation, most treatment cultures had high linolenic acid concentrations; nevertheless, according to the EN-14214 criteria, which specify a limit of 12 % for high-quality biodiesel, special attention should be paid to linolenic acid.

### Fuel properties

The composition of fatty acids predominantly determines the qualities of fuel. Typically, the Cetane Number (CN), heat of combustion, and viscosity augment with increasing chain length, indicating a preference for long-chain fatty acids (C16–C18) [60]. The CN increases in fuels with a high content of saturated fatty acids [65] an increase in CN correlates with enhanced ignition quality. Saturated esters possess a high and advantageous cetane number, and they have subpar cold flow properties. Unsaturated, particularly polyunsaturated fatty esters have lower melting points and are advantageous for enhanced low-temperature characteristics; nevertheless, they also possess low cetane numbers and diminished oxidative stability, which are detrimental for fuel [66]. Consequently, an appropriate balance of saturated and unsaturated FAMEs is essential for superior biodiesel quality [67]. This study compared the properties of *T. obliquus* biodiesel to American (ASTM D-6751) and European standards (EN 14214). CN readings range from 61.52 to 72.29, surpassing the requisite minimum value of 51.34 established by international standards [66], the previous result suggested that *T. obliquus* biodiesel’s CN values have superior ignition properties and should perform efficiently in engines according to both the American and European standards.

This investigation’s saponification value (SV) ranged from 169.16 to 232.51 mg/gm KOH. These findings were consistent with a recent study in *T. obliquus*, which found that SV ranged from 93.64 to 217.50 mg/gm KOH [2, 60, 68]; as the variation in SV can be attributed to differences in the fatty acid composition, which is influenced by the algae’s growth conditions, and the heavy metals concentration and type. The fatty acid molecular weight is inversely proportional to the saponification value [59]. The SV limit [69] was not defined by international biodiesel standards such as ASTM D-6751, EN 14214, or IS 15607. The amount of unsaturation in FAs is governed by the iodine value (IV), which increases the number of double bonds in the fatty acid chain and exclusively relies on the oil’s origin [63].

High iodine value levels can make biodiesel more vulnerable to oxidative assault, cause deposits, and reduce lubricity [60]. The results showed that all of the treated *T. obliquus* cultures had iodine values ranging from 15.75 to 66.86 g I_2_/100 g oil, which are less than the maximum limit of IV biodiesel requirements established by ASTM D-6751, EN 14214, and IS 15607—notably, the lowest iodine value correlates to biodiesel with the lowest unsaturated fatty ester content, as the lowest iodine value results from a shift in fatty acid composition towards more saturated fatty acids, inhibition of desaturase enzymes that prioritizes the production of more stable lipids [60]. A recent study found similar lower iodine value for *S. obliquus*, ranging from 40.97 to 68.20 I_2_/100 g oil [70].

Biodiesel oxidative stability depends on degree of unsaturation (DU) [60]. High PUFA concentrations reduce biodiesel viscosity and improve mechanical fuel characteristics. FAMEs from *T. obliquus* have DUs from 18.71 to 70.74 (wt.%), below the European regulatory limit of 137 wt.% [63].

The earlier study found DU varied from 67.80 to 94.09 (wt. %) [60]. Low-temperature biodiesel flow is a significant application challenge. PP is the lowest temperature at which biodiesel flows [71]; hence, it is used to quantify flow characteristics, as PP is higher in biodiesel made from saturated fatty acids [72]. The PP of this study’s biodiesel varies from −1.10 to 21.26 °C, meeting ASTM (IS 15607) criteria. It’s best for mild-climate areas. *T. obliquus* biodiesel had a higher flash point (FP) than ASTM D6751 (120 °C) and Petro diesel. The heavy metal exposure leads to the production of more saturated fatty acids and longer-chain FAMEs, which are less volatile and more stable, resulting in a higher flash point, As higher flash points indicate less flammable fuel, handling, storage, and transport are safer [73].

The oxidative stability (OS) of FAMEs in regarded to *T. obliquus* throughout this investigation ranged from 5.05 to 7.05 h. This range aligns with the ASTM D6751 requirement of 3 h and the EN 14214 recommendation of 6 h. The variation in OS can be attributed to the low polyunsaturated fatty acids content. The elevated levels of PUFA in biodiesel lead to rapid oxidation, negatively impacting storage stability, a critical factor for fuel applications [74]. Prior studies have identified an inverse correlation between oxidation stability and the degree of unsaturation, particularly in the case of polyunsaturated fatty acids (C18:2, C18:3)[63]; this is due to the presence of reactive sites in unsaturated fatty acid chains, which are particularly susceptible to free radical approach [74].

## Conclusion

This study highlights the potential of using *T. obliquus* under heavy metal stress conditions as a sustainable approach to optimize lipid yield and fatty acid profiles for biodiesel production. Our findings suggest that *T. obliquus* can be a viable candidate for biodiesel production when cultivated under specific heavy metal concentrations. Optimizing lipid yield and biodiesel quality metrics is possible by strategically manipulating heavy metal stress. Overall, while heavy metal stress shows promise in controlled laboratory settings, applying it at an industrial scale remains challenging due to environmental and regulatory barriers. For these reasons, exploring alternative stressors (e.g., nutrient deprivation or salinity stress) might be more practical for enhancing lipid productivity in large-scale algal cultivation. Future work should focus on refining lipid extraction methods, specifically targeting the removal of any contaminants to ensure biodiesel quality. Finally, long-term studies are required to evaluate the genetic stability and performance of *T. obliquus* cultures under continuous, industrial-scale conditions to ensure the feasibility of this approach for commercial production.

## Data Availability

Data is provided within the manuscript or supplementary information files.
